# Outcomes of robotic-assisted surgery for pediatric renal tumors: a systematic review

**DOI:** 10.1007/s11701-025-02473-4

**Published:** 2025-06-20

**Authors:** Amani N. Alansari, Mohmed Sayed Zaazouee, Alaa Ahmed Elshanbary

**Affiliations:** 1 Department of Pediatric Surgery, Hamad Medical Corporation, P.O Box: 3050, Doha, Qatar; 2 Faculty of Medicine, Al-Azhar University, Assiut, Egypt; 3 Faculty of Medicine, Alexandria University, Alexandria, Egypt

**Keywords:** Robotic-assisted surgery, RAS, Renal tumors, Pediatric, Wilms tumor, Systematic review

## Abstract

Robotic-assisted surgery (RAS) has gained interest due to its precision and minimally invasive advantages. However, its adoption in pediatric renal tumors remains limited, with concerns about feasibility, safety, and oncological outcomes. This systematic review summarized the literature evaluating the outcomes of RAS for pediatric renal tumors. We searched PubMed, Scopus, and Web of Science for studies on RAS in pediatric renal tumors. We considered all primary studies including case reports, case series, and cohort studies. Quality was assessed using Joanna Briggs Institute (JBI) critical appraisal tools. A total of 14 studies, including 7 case reports, 5 case series, and 2 cohort studies, with 79 pediatric patients undergoing RAS for renal tumors were included. Conversion to open surgery occurred in 6.3% of cases. Renal vein injury was reported in 2.5%. Operative times ranged from 90 to 360 min, with shorter durations for partial nephrectomies. Blood loss was highly variable, from < 5 mL in smaller tumors to > 350 mL in complex cases. Hospital stays ranged from 2 to 7 days, with one outlier requiring a 4-week hospitalization. Oncological outcomes were generally favorable, with 96.2% of patients remaining disease free at follow-up. However, three patients (3.8%) experienced local recurrence, and two patients (2.5%) with WT died due to CNS metastases. This systematic review demonstrates that RAS is a feasible and safe approach for managing pediatric renal tumors. However, larger studies with extended follow-up are necessary to validate long-term oncological outcomes and refine patient selection criteria.

**Supplementary Information:**

The online version contains supplementary material available at 10.1007/s11701-025-02473-4.

## Introduction

Renal tumors, while among the most frequently diagnosed solid tumors in children, account for only approximately 3 to 10% of all pediatric malignancies [1]. Wilms tumor (WT) or nephroblastoma accounts for the majority of that number, spanning 80% to 85% of all renal tumors in children. Other types including renal cell carcinoma, mesoblastic nephroma, clear cell sarcoma, and rhabdoid tumor are significantly rare, accounting for only 2 to 4% of the cases [2]. The management of pediatric renal tumors, particularly WT, has been guided by two major collaborative research groups: the International Society of Pediatric Cancer (SIOP) and the Children’s Oncology Group (COG) [3, 4]. SIOP applies preoperative chemotherapy, while COG favors immediate surgery together with adjuvant therapy. Despite this disagreement between groups, there are over 90% survival rates for localized WT in both of them [5].

Over the past 2 decades, minimally invasive surgeries (MIS), particularly laparoscopic and robotic-assisted surgery (RAS), have been shown to be effective in managing localized renal cell carcinoma in adults [6–8]. They demonstrate superiority over traditional open surgical approaches due to their association with reduced intraoperative blood loss, decreased postoperative morbidity, shorter hospitalization duration, and expedited recovery. RAS represents the latest advancement in minimally invasive surgical techniques. It integrates principles of medicine, robotics, and engineering to achieve unparalleled surgical precision and outcome [9]. It has gained widespread interest over conventional laparoscopy due to its enhanced dexterity, and three-dimensional visualization [10, 11]. However, these advantages come at a significantly higher cost, which remains a major consideration in its broader adoption [12].

The integration of RAS in pediatric surgery is gaining interest but remains limited due to several challenges [13]. The restricted space within the small abdominal cavity of children presents considerable challenges for instrument maneuverability. Moreover, pediatric surgeons often face a steep learning curve when transitioning from traditional open or laparoscopic methods to robotic techniques [14–16]. While RAS is well-established in adult renal oncology, its adoption in pediatric cases has been slower, partly due to the low incidence of pediatric renal tumors, complex tumor presentations, and a lack of widespread robotic expertise among pediatric surgeons [17]. There are also doubts surrounding oncological safety regarding possible tumor spillage and incompleteness of resection, which has posed hesitancy in widespread implementation [18]. Despite these challenges, some reports have demonstrated the feasibility of RAS in managing renal tumors [19, 20]. The current literature on RAS for pediatric renal tumors remains limited, with a lack of large-scale studies and standardized protocols. This systematic review aims to assess the existing evidence on RAS in this context, focusing on surgical outcomes, perioperative complications, and oncologic efficacy. By synthesizing data from case reports, case series, and cohort studies, it evaluates the feasibility and safety of robotic-assisted nephrectomy in children while highlighting its potential advantages and identifying key areas for future research.

## Materials and methods

This systematic review was conducted in accordance with the Preferred Reporting Items for Systematic reviews and Meta-Analyses (PRISMA) guidelines [21].

### Search strategy

We searched PubMed, Scopus, and Web of Science (WOS) to identify relevant studies on robotic-assisted surgery for pediatric renal tumors. These search terms were employed: robotic surgical procedures, robotic, robot-assisted, robotic-assisted, minimally invasive surgery, robot-assisted partial nephrectomy (RAPN), kidney neoplasms, renal tumor, kidney tumor, Wilms tumor, Wilms’ tumor, nephroblastoma, renal mass, pediatrics, child, pediatric, adolescents, infant, and young patients. Details of search strategies and search results across the three databases are demonstrated in *Supplementary Table 1*. The retrieved records underwent a two-step screening process by two independent reviewers: initially through title and abstract screening, followed by full-text screening. Any disagreements between the reviewers were resolved through discussion, and if consensus was not reached, a third reviewer was consulted.

### Eligibility criteria

Studies were considered based on the population, intervention, control, outcomes and study design (PICOS) framework: (1) Population: pediatric patients under the age of 18 with renal tumors, (2) Intervention: RAS for partial or radical nephrectomy, (3) Control: both single-arm and controlled studies comparing RAS with other approaches, (4) perioperative, and/or oncological outcomes, and (5) Study design: all primary studies, including case reports, case series, and cohort studies, were included due to the limited literature in this area. Studies were excluded if they focused on conventional laparoscopy, adult populations, reviews, editorials, or conference abstracts without relevant data. Only full-text articles published in English were included.

### Data extraction and quality assessment

Relevant data from the included studies were systematically extracted through a pre-defined data extraction form. Collected variables include study characteristics (study design, year of publication, country), patient demographics (number of patients, age, gender), and clinical information (primary diagnosis, neoadjuvant chemotherapy and tumor size in millimeters). Surgical details were also recorded including type of robotic-assisted procedure performed, operation time in minutes, blood loss in milliliters, and conversion to open surgery. Postoperative variables, including hospital stay duration (days), complication rates, and follow-up period (months), were assessed, along with oncologic outcomes such as local recurrence and overall survival (if reported). All data were independently verified for accuracy and consistency by two independent reviewers.

The Joanna Briggs Institute (JBI) tools were used to evaluate the quality of the included studies [22]. Three specific checklists were applied for case reports, case series, and cohort studies. Key evaluation criteria included study design, patient selection, outcome assessment, and statistical analysis to determine reliability and risk of bias [23].

## Results

### Search results

**Fig. 1:**
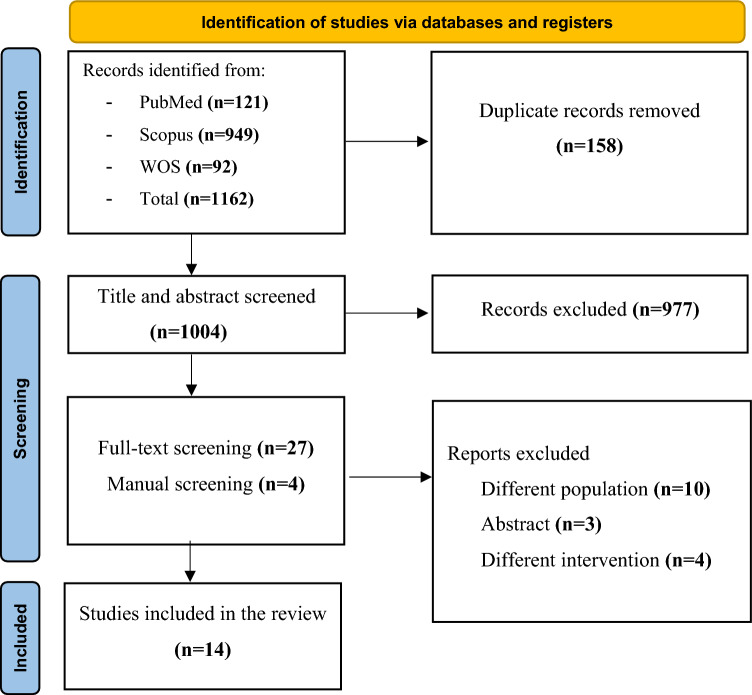
The PRISMA flowchart

The database search yielded a total of 1162 records. After removing 158 duplicates, 1004 studies remained. Following the title and abstract screening, 27 full-text articles were assessed for eligibility based on the inclusion criteria. Finally, 14 studies met the inclusion criteria. The PRISMA flowchart is demonstrated in Fig. 1.

### General characteristics

A total of 14 studies with a total of 79 subjects were included: 7 case reports [24–30], 5 case series [31–35], and 2 cohort studies [19, 20]. The studies focus predominantly on pediatric renal tumors, with Wilms tumor being the most common diagnosis, followed by rarer entities like clear cell renal cell carcinoma (RCC), metanephric adenoma, tubulopapillary carcinoma, undifferentiated sarcoma, and metanephric stromal tumor. Neoadjuvant chemotherapy was frequently administered according to SIOP protocols in WT cases, with regimens varying between Vincristine, Dactinomycin (AV), and more intensive combinations including Doxorubicin (AVD). In total, 67 cases received neoadjuvant chemotherapy, while 12 did not. Notably, 8 of these 12 cases were non-Wilms tumors, which typically follow different treatment protocols, with surgical resection often being the primary approach. The procedures included both radical nephrectomy and nephron-sparing surgery (partial nephrectomy). Tumor sizes showed considerable variability, ranging from 2.7 mm in nephron-sparing cases to 980 mm in aggressive sarcomas. A summary of the included studies is demonstrated in Table 1.

**Table 1:** Summary of the included studies

Author	Study design	Number	Age	Gender	Diagnosis	Neoadjuvant chemotherapy	Procedure	Tumor size (mm)	Operation time (minutes)	Length of hospital stay (day)	Follow-up (months)	Conversion or Complications	Local recurrence; overall survival	Blood loss (mm)
He 2024 [19]	Retrospective study	17	6 ± 3	8 F:9 M	9 Wilms’ tumors 5 RCC 3 metanephric adenomas	Based on SIOP protocol	Radical nephrectomy: 14 Partial nephrectomy: 3	The maximum tumor diameter: 55.33 ± 16.5	177 ± 22.5	4.5 ± 2	29	NR	1 recurrence (Wilms tumor Stage II, 6 M); 100% survival	23.8 ± 11.3
Li 2024 [35]	Case series	12	3.8	5 F:7 M	Wilms tumor	AV (Vincristine, Dactinomycin)	Radical nephrectomy	Tumor volume:135 mL	NSS: 72.5 ± 3.82 RN: 98.17 ± 12.47 RN + IVCT: 320	NR	18.5 ± 5.5	NR	No recurrence or cancer-related deaths	NSS: 27 ± 4 RN: 41.67 ± 12.13 RN + IVCT: 350
			1.5		Wilms tumor	AVD (Vincristine, Dactinomycin, Doxorubicin)	Radical nephrectomy	Tumor volume:136.5 mL						
			8.6		Wilms tumor	Rejected	Radical nephrectomy	Tumor volume:704 mL						
			4.8		Wilms tumor	None	Radical nephrectomy	Tumor volume:9.4 mL						
			1.7		Wilms tumor	AV (Vincristine, Dactinomycin)	Radical nephrectomy	Tumor volume:55.25 mL						
			2.2		Wilms tumor	AVD (Vincristine, Dactinomycin, Doxorubicin)	Radical nephrectomy	Tumor volume:55.72 mL						
			1.3		Wilms tumor	AVD (Vincristine, Dactinomycin, Doxorubicin)	Radical nephrectomy	Tumor volume:4.49 mL						
			1.2		Wilms tumor	None	Nephron-sparing surgery	Tumor volume:2.7 mL						
			2.9		Wilms tumor	AVD (Vincristine, Dactinomycin, Doxorubicin)	Nephron-sparing surgery	Tumor volume:6 mL						
			1.8		Wilms tumor	None	Nephron-sparing surgery	Tumor volume:3.5 mL						
			2.5		Wilms tumor	None	Nephron-sparing surgery	Tumor volume:11.52 mL						
Della Corte 2023 [30]	Case report	1	4	Female	Wilms tumor	No	Partial nephrectomy	41 × 37 × 41	180	4	28	NR	None; alive	20–30
Van Der Jeugt 2023 [34]	Case series	2	4	NR	Wilms tumor	Yes (UMBRELLA/SIOP)	Radical nephrectomy	Pre-chemotherapy: 83 × 57 × 76 Post-chemotherapy: 61 × 61 × 55	200	4	6	NR	None; tumor-free margins	10
			3		Wilms tumor		Radical nephrectomy	Pre-chemotherapy: 55 × 55 × 54 Post-chemotherapy: 35 × 28 × 24	95	3	6	NR	None; tumor-free margins	5
**Blanc 2022 **[20]	Prospective observational study	24	8.25 ± 2.35	NR	20 Wilms tumors 1 renal undifferentiated sarcoma 1 metanephric adenoma 1 nephrogenic rest 1 tubulopapillary carcinoma	- 20 Wilms tumors received neoadjuvant chemotherapy - 1 renal undifferentiated sarcoma received neoadjuvant chemotherapy	18 radical nephrectomy 6 partial nephrectomies (retroperitoneal approach)	NR	217 ± 31.5	3 ± 0.5	29.25 ± 5.75	5 conversions to open surgery occurred in renal tumors 1 emergency undocking due to renal vein bleeding	1 patient with Wilms tumor had a local recurrence with massive pleural recurrence, currently on second-line chemotherapy 1 patient with bilateral nephroblastomatosis had recurrence twice, managed with open surgery, chemotherapy, and radiotherapy 1 girl with Wilms tumor died from central nervous system metastasis	NR
**Sala 2020 **[28]	Case report	1	3	Male	Bilateral Wilms tumor	Yes (SIOP-UMBRELLA-2016)	Right partial nephrectomy + left radical nephrectomy	NR	90	2	NR	NR	NR	50
**Silveri 2020 **[29]	Case report	1	14	Male	Atypical renal perivascular epithelioid cell tumor	No	Partial nephrectomy	41 × 32	NR	3	8	NR	None at 8 months	NR
**Blanc 2019 **[33]	Case series	10	5.1	7 F:3 M	Wilms tumor	Given according to SIOP-2001 protocol for Wilms tumor, intensified chemotherapy for some cases	Radical nephrectomy	Tumor volume: 172 mL	120	4	25	Renal vein injury	None; alive	NR
			5.2		Wilms tumor		Radical nephrectomy	Tumor volume:240 mL	180	2	20	NR	None; alive	
			4.2		Wilms tumor		Radical nephrectomy	Tumor volume:142 mL	270	5	17	Difficult renal hilum dissection (renal sarcoma)	Dead due to CNS metastases	
			3.6		Wilms tumor		Radical nephrectomy	Tumor volume:164 mL	220	7	15	NR	None; alive	
			4.7		Wilms tumor		Partial nephrectomy	NR	120	4	14	NR	None; alive	
			3.2		Wilms tumor		Partial nephrectomy	NR	110	4	13	NR	None; alive	
			14.1		Tubulopapillary carcinoma		Partial nephrectomy	NR	140	2	12	NR	None; alive	
			10.1		Wilms tumor		Radical nephrectomy	Tumor volume:814 mL	360	4	11	NR	None; alive	
			12.8		Sarcoma		Radical nephrectomy	Tumor volume:225 mL	145	4	8	Misdiagnosed renal vein tumor thrombus	None; alive	
			3.5		Wilms tumor		Radical nephrectomy	Tumor volume: 212 mL	360	4	4	NR	None; alive	
**Tiryaki 2019 **[27]	Case report	1	8	Female	Metanephric stromal tumor	No	Partial nephrectomy	35 × 25	160	3	12	NR	None; alive	20
**Meignan 2018 **[32]	Case series	2	3	Female	Wilms tumor	Yes (SIOP protocol)	Radical nephrectomy	2 × 2.2 × 1	263	4	74	NR	None; alive	< 5
			12	Male	Metanephric adenoma	No	Partial nephrectomy	2.3 × 1.8 × 1.9	105	NR	23	NR	None; alive	< 5
**Varda 2018 **[31]	Case series	4	13.5 ± 9	NR	Papillary RCC (AJCC pT1aNx) and two cases of segmental cystic renal dysplasia with nephrogenic rests	No	Partial nephrectomies with retroperitoneal lymph node dissection	Mass size: 258 ± 94	290 ± 34	4.3 ± 1.3	NR	NR	None; alive	5–300
		1	19	NR	Undifferentiated sarcoma	No	Nephrectomy with pericaval LND	Mass size: 980	234	3	NR	NR	None; alive	250
**Yadav 2018 **[26]	Case report	1	1.5	Female	Wilms tumor	Yes (SIOP protocol)	Partial nephrectomy	Pre-treatment: 33 × 23 × 22 mm Post-neoadjuvant: 19 × 14 × 10 mm	NR	28	At least 1.5 months (drain removed at 4 weeks, DJ stent at 6 weeks)	Persistent drain output for 4 weeks	None; preserved renal function	NR
**Cost 2015 **[25]	Case report	1	14	Female	Wilms tumor, Stage II	No	Radical nephrectomy	60 × 50 × 35	210	2	NR	NR	No recurrence reported; ongoing treatment with EE-4A	NR
**Cost 2012 **[24]	Case report	1	14	Female	Clear-cell RCC, AJCC stage T1aN0M0	No	Partial nephrectomy with extended lymph node dissection (aortic and hilar)	15	180	2	6	NR	None; surveillance ongoing per AREN0321	NR

*RCC* renal cell carcinoma, *AJCC* American Joint Committee on Cancer, *NSS* nephron-sparing surgery, *RN* radical nephrectomy, *RN* + *IVCT* radical nephrectomy + inferior vena cava thrombectomy, *NR* not reported

### Surgical outcomes

Surgical conversion to open surgery was documented in five cases (6.3%). Renal vein injury occurred in two cases (2.5%). Operative times varied across cases, with radical nephrectomies typically ranging from 90 to 360 min, while nephron-sparing surgeries were associated with shorter operative durations. Blood loss was inconsistent, with minimal loss (< 5 mL) in smaller tumors and exceeding 350 mL in more complex cases. Hospital stay durations were generally short, varying from 2 to 7 days. However, one case was reported to stay for 4 weeks [26]. This was due to persistent drain output, necessitating extended hospitalization. Complication rates differed, with some studies reporting no major postoperative events, while others documented renal vein bleeding, and challenging hilar dissections. One case of misdiagnosed renal vein thrombus mandated conversion to open surgery. One patient required emergency robotic undocking due to intraoperative renal vein bleeding. The comparative study by He et al. demonstrated that patients undergoing RAS experienced significantly lower intraoperative blood loss and a shorter postoperative hospital stay compared to those who underwent laparoscopic surgery [19].

### Oncological outcomes

The follow-up period varied among studies, ranging from as short as 1.5 months to as long as 74 months. However, most studies followed patients for a duration between 6 months and 2 years, providing insights into mid-term outcomes. Overall, oncological outcomes were favorable, with the majority of patients remaining disease-free during the follow-up period. Nevertheless, three cases (3.8%) experienced local recurrence. Two patients with Wilms tumor ultimately died due to CNS metastases.

### Quality assessment

The quality assessment using the JBI critical appraisal tools showed that most case reports and series were rated as high quality, scoring 7/8 to 9/10. Only a few case series received moderate ratings due to unclear reporting in certain domains. Among the cohort studies, He et al. was classified as high quality, whereas Blanc et al. was rated as low due to several unclear or negative assessments. The included studies exhibited predominantly high methodological quality, although they were limited by some issues in reporting and study design. Table 2, 3, and 4 demonstrates the quality assessment of the included studies, with sections A, B, and C covering case reports, case series, and cohort studies, respectively.

**Table 2:** Quality assessment using JBI Critical Appraisal Checklist: case reports

ID	Q1	Q2	Q3	Q4	Q5	Q6	Q7	Q8	Total	Quality
Della Corte 2023 [30]	Y	Y	Y	Y	Y	Y	N	Y	7/8	High
Sala 2020 [28]	Y	Y	Y	Y	Y	Y	N	Y	7/8	High
Silveri 2020 [29]	Y	Y	Y	Y	Y	Y	N	Y	7/8	High
Tiryaki 2019 [27]	Y	Y	Y	Y	Y	Y	N	Y	7/8	High
Yadav 2018 [26]	Y	Y	Y	Y	Y	Y	Y	Y	8/8	High
Cost 2015 [25]	Y	Y	Y	Y	Y	Y	N	Y	7/8	High
Cost 2012 [24]	Y	Y	Y	Y	Y	Y	N	Y	7/8	High

*Y* yes, *N* no

Q1: Were patient’s demographic characteristics clearly described?

Q2: Was the patient’s history clearly described and presented as a timeline?

Q3: Was the current clinical condition of the patient on presentation clearly described?

Q4: Were diagnostic tests or assessment methods and the results clearly described?

Q5: Was the intervention(s) or treatment procedure(s) clearly described?

Q6: Was the post-intervention clinical condition clearly described?

Q7: Were adverse events (harms) or unanticipated events identified and described?

Q8: Does the case report provide takeaway lessons?

**Table 3:** Quality assessment using JBI Critical Appraisal Checklist: case series

ID	Q1	Q2	Q3	Q4	Q5	Q6	Q7	Q8	Q9	Q10	Total	Quality
Li 2024 [35]	Y	Y	Y	U	U	Y	Y	Y	N	NA	6/10	Moderate
Van Der Jeugt 2023 [34]	Y	Y	Y	U	U	Y	Y	Y	N	NA	6/10	Moderate
Blanc 2019 [33]	Y	Y	Y	U	U	Y	Y	Y	Y	Y	8/10	High
Meignan 2018 [32]	Y	Y	Y	U	U	Y	Y	Y	Y	N	7/10	Moderate
Varda 2018 [31]	Y	Y	Y	U	Y	Y	Y	Y	Y	Y	9/10	High

*Y* yes, *N* no, *U* unclear, *NA* not applicable

Q1: Were there clear criteria for inclusion in the case series?

Q2: Was the condition measured in a standard, reliable way for all participants included in the case series?

Q3: Were valid methods used for identification of the condition for all participants included in the case series?

Q4: Did the case series have consecutive inclusion of participants?

Q5: Did the case series have complete inclusion of participants?

Q6: Was there clear reporting of the demographics of the participants in the study?

Q7: Was there clear reporting of clinical information of the participants?

Q8: Were the outcomes or follow-up results of cases clearly reported?

Q9: Was there clear reporting of the presenting site(s)/clinic(s) demographic information?

Q10: Was statistical analysis appropriate?

**Table 4:** Quality assessment using JBI Critical Appraisal Checklist: cohort studies

Authors	Q1	Q2	Q3	Q4	Q5	Q6	Q7	Q8	Q9	Q10	Q11	Total	Quality
He 2024 [19]	Y	Y	Y	N	N	Y	Y	Y	U	Y	Y	8/11	High
Blanc 2022 [20]	NA	NA	Y	U	Y	N	Y	Y	U	U	Y	5/11	Low

*Y* yes, *N* no, *U* unclear, *NA* not applicable

Q1: Were the two groups similar and recruited from the same population?

Q2: Were the exposures measured similarly to assign people to both exposed and unexposed groups?

Q3: Was the exposure measured in a valid and reliable way?

Q4: Were confounding factors identified?

Q5: Were strategies to deal with confounding factors stated?

Q6: Were the groups/participants free of the outcome at the start of the study (or at the moment of exposure)?

Q7: Were the outcomes measured in a valid and reliable way?

Q8: Was the follow-up time reported and sufficient to be long enough for outcomes to occur?

Q9: Was follow-up complete, and if not, were the reasons for loss to follow-up described and explored?

Q10: Were strategies to address incomplete follow-up utilized?

Q11: Was appropriate statistical analysis used?

## Discussion

This systematic review on RAS in pediatric renal tumors explores the expanding application of this technique in the management of renal tumors in children. The findings of this study indicate that RAS is a feasible and safe approach, with an acceptable rate of surgical complications. Oncological outcomes were good, with only three cases of local recurrence (3.8%), and two deaths due to CNS metastases. It supports growing experiences in adopting minimally invasive techniques in pediatric oncology and at the same time underscore the need for much higher-quality studies to prove the long-term oncological safety of RAS, especially in nephron-sparing approaches.

The first case of RAS for a pediatric renal tumor was reported in 2012, describing the feasibility of robotic partial nephrectomy for renal cell carcinoma (RCC) in a 14-year-old female, emphasizing its potential for tumor resection and extended lymph node dissection [24]. Three years later, in 2015, the first case of robotic-assisted radical nephrectomy for WT was reported in a female of similar age, highlighting the application of RAS for more extensive procedures [25]. Subsequent reports expanded its application to involve much younger children [28, 32, 34, 35]. Yadav et al. described the first use of RAS in an infant as young as 1.5 years old [26]. This highlights its feasibility across different pediatric age groups.

Surgical and oncological outcomes varied across the cases based on variable factors that include tumor type, size, location, vascular involvement, and preoperative chemotherapy response. Conversions to open surgery occurred due to vascular injury (renal vein injury or misdiagnosed thrombus), difficult renal hilum dissection, mechanical failure (sliding clip), and respiratory challenges [20, 33]. Notably, all conversions were reported in studies by Blanc et al., potentially reflecting differences in surgical expertise at the center or the possible inherent complexity of the cases. The overall conversion rate for renal tumors in our review was 6.3%, higher than reported rates for adult renal surgeries [36]. Hospital stays were generally brief, except for the youngest patient (1.5 years old), who required a 4-week stay due to persistent drain output, perhaps from lymphatic leakage or incomplete hemostasis [26]. The local recurrence rate following RAS was 3.8%, comparable to the reported rate in laparoscopic resection and lower than the 15% reported for open surgery [37, 38]. However, longer follow-up is required to validate these findings.

To optimize surgical outcomes and minimize complications, careful patient selection for RAS is crucial. Blanc et al. proposed guidelines based on their study finding [20]. The recommended criteria include: tumors confined within the ipsilateral spinal border, a thick rim of normal renal parenchyma, no evidence of extrarenal infiltration, and an estimated tumor volume-to-effective postoperative blood volume (ETV/EPBV) ratio of less than 1.5%. Cases with a thinner parenchymal rim, tumors extending beyond the ipsilateral spinal border but not crossing the midline, or an ETV/EPBV ratio between 1.5% and 2% may still be considered with caution. In contrast, tumors that cross the midline, infiltrate extrarenal structures such as the liver or diaphragm, encase renal vessels, or have an ETV/EPBV ratio above 2% present significant surgical challenges and are less suitable for RAS.

He et al. conducted a comparative analysis of RAS versus laparoscopic-assisted surgery (LAS) for pediatric renal tumors [19]. Among 23 patients (RAS: 17, LAS: 6), RAS was associated with less blood loss and shorter hospital stays, while operative times and oncologic outcomes were similar. Two LAS cases required conversion to open surgery due to bleeding, whereas none did in the RAS group. However, the study’s small sample size and unmatched groups limit its conclusions.

The gold standard for managing WT has been radical nephrectomy by the transperitoneal approach. Nephron-sparing surgery (NSS) is primarily considered for bilateral disease or solitary kidneys to preserve renal function. Due to the long-term effects of chronic kidney disease, hypertension, and cardiovascular complications, NSS is recommended for unilateral non-syndromic WT when technically feasible. However, keeping tight patient eligibility and management at specialized centers is critical for effective outcomes. In 2018, Yadav et al. reported the first case of robotic NSS in a child with syndromic WT [26]. In 2020, Sala et al. reported a case of bilateral WT undergoing RAS, involving a right partial nephrectomy and a left radical nephrectomy [28]. The protocol developed by the Renal Tumor Study Group of the International Society of Pediatric Oncology (SIOP–RTSG) permits NSS for solitary, non-syndromic WTs, provided the tumor is smaller than 300 mL, there is no lymph node involvement, and the remaining kidney is expected to function well after preoperative chemotherapy [39]. In 2023, Della Corte et al. reported the first case of transperitoneal robot-assisted partial nephrectomy for a unilateral, non-syndromic WT. They worked on a child of 4 years of age with incidentally-diagnosed renal mass measuring 3.6 cm. At 28 months of follow-up, no tumor recurrence was detected [30].

This systematic review has several limitations. First, with most of the studies being case reports and small case series, it is difficult to draw firm conclusions regarding the safety and long-term oncological effects of RAS in pediatric renal tumors. Second, the heterogeneity in study designs, surgical techniques, tumor characteristics, and follow-up durations further complicates consistent conclusions. Finally, the influence of small sample sizes and variations in neoadjuvant chemotherapy protocols on surgical and oncological outcomes highlights the need for larger prospective studies. We recommend further investigation and research to better define the role of RAS in unilateral non-syndromic WT. Moreover, comparative studies are warranted to establish a standardized assessment of RAS against conventional laparoscopic or open approaches. Given the significant cost associated with RAS, future research should also include cost-effectiveness analyses to determine its viability in routine clinical practice. Eventually, long-term oncological follow-up and functional renal outcomes should be explored to better define the cons and pros of RAS in pediatric renal tumors.

## Conclusion

Robotic-assisted surgery in pediatric renal tumors appears to be feasible and promising, with advantages including less intraoperative blood losses, shorter hospital stays, and oncological results. According to this systematic review, RAS can be performed safely for radical and nephron-sparing approaches in selected cases, with minimal complications and recurrence. In spite of that, its applications are still limited due to high cost, technical challenges, especially in young children, in addition to the steep robot surgical-learning curve. Future research should include multicenter prospective studies with larger cohorts and standardized protocols, so that the long-term oncological outcomes of RAS can be better defined. Comparative studies assessing functional outcomes, recurrence rates, and survival data between robotic, laparoscopic, and open approaches will be crucial in defining the role of RAS in pediatric renal oncology. Though RAS presents a compelling alternative, its use should remain selective and guided by careful patient assessment until more robust evidence emerges.

## Supplementary Information

Below is the link to the electronic supplementary material.

Supplementary file1 (DOCX 18 KB)

## Data Availability

No datasets were generated or analysed during the current study.
