# Supplementary material for: Outcomes of robotic-assisted surgery for pediatric renal tumors: a systematic review
Source: J Robot Surg. 2025 Jun 20;19(1):310. doi: 10.1007/s11701-025-02473-4 (PMC12181127; doi:10.1007/s11701-025-02473-4)
Supplement: Supplementary file 1 — Supplementary file1 (DOCX 18 KB) [file 11701_2025_2473_MOESM1_ESM.docx]

| **Database** | **Search strategy** | **Number of results** |
| --- | --- | --- |
| **PubMed** | ("Robotic Surgical Procedures"[Mesh] OR robotic OR robot-assisted OR robotic-assisted OR "minimally invasive surgery" OR "RAPN") AND ("Kidney Neoplasms"[Mesh] OR "renal tumor" OR "kidney tumor" OR "Wilms tumor" OR "Wilms’ tumor" OR "nephroblastoma" OR "renal mass") AND ("Pediatrics"[Mesh] OR "child*" OR "pediatric" OR "adolescents" OR "infant" OR "young patients") | **121** |
| **SCOPUS** | (TITLE-ABS-KEY("Robotic Surgical Procedures" OR robotic OR robot-assisted OR robotic-assisted OR "minimally invasive surgery" OR "RAPN") AND ("Kidney Neoplasms" OR "renal tumor" OR "kidney tumor" OR "Wilms tumor" OR "Wilms’ tumor" OR "nephroblastoma" OR "renal mass") AND ("Pediatrics" OR "child*" OR "pediatric" OR "adolescents" OR "infant" OR "young patients")) | **949** |
| **Web of Science** | ("Robotic Surgical Procedures"[Mesh] OR robotic OR robot-assisted OR robotic-assisted OR "minimally invasive surgery" OR "RAPN") AND ("Kidney Neoplasms"[Mesh] OR "renal tumor" OR "kidney tumor" OR "Wilms tumor" OR "Wilms’ tumor" OR "nephroblastoma" OR "renal mass") AND ("Pediatrics"[Mesh] OR "child*" OR "pediatric" OR "adolescents" OR "infant" OR "young patients") | **92** |

**Supplementary Table 1.** Search strategy and search results.
